# Quantitative association between lead exposure and amyotrophic lateral sclerosis: a Bayesian network-based predictive study

**DOI:** 10.1186/s12940-023-01041-3

**Published:** 2024-01-03

**Authors:** Wenxiu Yu, Fangfang Yu, Mao Li, Fei Yang, Hongfen Wang, Han Song, Xusheng Huang

**Affiliations:** 1 Medical School of Chinese PLA, Beijing, 100853 China; 2 Neurological Department of the First Medical Center, Chinese PLA General Hospital, Beijing, 100853 China; 3 Department of Medical Innovation Research, PLA General Hospital, Beijing, 100853 China; 4 Department of Health Service, Chinese PLA General Hospital, Beijing, 100853 China

**Keywords:** Pb exposure, Amyotrophic Lateral Sclerosis, Bayesian network

## Abstract

**Background:**

Environmental lead (Pb) exposure have been suggested as a causative factor for amyotrophic lateral sclerosis (ALS). However, the role of Pb content of human body in ALS outcomes has not been quantified clearly. The purpose of this study was to apply Bayesian networks to forecast the risk of Pb exposure on the disease occurrence.

**Methods:**

We retrospectively collected medical records of ALS inpatients who underwent blood Pb testing, while matched controlled inpatients on age, gender, hospital ward and admission time according to the radio of 1:9. Tree Augmented Naïve Bayes (TAN), a semi-naïve Bayes classifier, was established to predict probability of ALS or controls with risk factors.

**Results:**

A total of 140 inpatients were included in this study. The whole blood Pb levels of ALS patients (57.00 μg/L) were more than twice as high as the controls (27.71 μg/L). Using the blood Pb concentrations to calculate probability of ALS, TAN produced the total coincidence rate of 90.00%. The specificity, sensitivity of Pb for ALS prediction was 0.79, or 0.74, respectively.

**Conclusion:**

Therefore, these results provided quantitative evidence that Pb exposure may contribute to the development of ALS. Bayesian networks may be used to predict the ALS early onset with blood Pb levels.

## Background

Amyotrophic lateral sclerosis (ALS), a fatal neurodegenerative disease, is characterized by progressive muscle weakness, swallowing difficulty, paralysis, and finally death within 2 to 5 years following diagnosis ( [1]). Currently, ALS progression cannot be cured or stopped. Causes of ALS are multifactorial: genetic mutations including Chromosome 9 Open Reading Frame 72 (C9ORF72), Superoxide Dismutase 1 (SOD1), or TAR DNA-binding Protein of 43 kDa (TDP-43) etc., account for 70% of familial ALS (fALS) and 15% of sporadic ALS (sALS), indicating environmental factors contribute to ALS risk and progression ( [2]). The interaction of genetic background with environmental exposures seemed likely underpin understanding of the disease onset ( [3]).

As a heavy metal without any physiological roles, lead (Pb) caused a wide range of toxic effects. Pb associated with several neurodegenerative diseases, like ALS, Alzheimer’s disease (AD), Parkinson’s disease (PD) ( [4]). A meta-analysis of 14 case-control studies found statistically higher Pb levels in ALS cases than controls in whole blood samples ( [5]). Our unpublished data provided novel evidence that Pb caused abnormal aggregation of SOD-1 in motor neurons by interfering with the chaperone functions of Glucose-Regulated Protein of 78 kDa (GRP78). We previously reported GRP78 played vital roles in regulating Pb-induced Src activation and its downstream of blood-brain barriers’ disruption ( [6]).

Early diagnosis of ALS was difficult because of its large heterogeneity in the clinical manifestations ( [7]). Current criteria established by the World Federation of Neurology recommended neurological and electrophysiological examinations for its diagnosis and antidiastole ( [8]). The diagnostic delay from ALS onset ranged from 6 to 21 months ( [9]). Accurate and early identification of the disease was crucial for providing personalized interventions, which helped prolong life expectancy and enhance the quality of life for ALS patients. However, whether Pb levels of human body will be used as a potential biomarker for ALS is still not clear.

Bayesian networks were used to understand the causal relationships in real-world probabilistic problems ( [10]). Bayesian networks has been considered as an efficient decision tool for predicting ALS disease in previous study ( [11]). Based on the medical big data system ( [12, 13]), we chose the ALS inpatients who underwent blood Pb testing as well as matched controlled inpatients admitted to the neurology department. Patient’s demographic information, vital signs, medical orders, examination reports, lab tests results were also obtained from the same study. In this study, we tried to (1) establish a risk prediction model by blood Pb concentrations combined with other factors using Bayesian networks, (2) examine the model performance with accuracy, specificity, and sensitivity.

## Methods

### Data collection

The medical records of ALS inpatients were collected admitted to military hospitals from November 2014 to October 2018. The information analyzed included general information, whole blood metal levels and related lab tests results. The Pb concentrations in the whole blood were measured by atomic absorption spectroscopy. This study was performed with historical data that removed private information; thus, it was exempt from Institutional Review Board approval.

### Data resource

The ALS standardized diagnosis (G12.2, motor neuron disease) under the International Disease Classifcation System ICD-10 was used as the retrieval basis. To date, blood Pb test was not routine practice for neurological patients. It was carried out when clinical symptoms associated with Pb exposure were present. Thus, the target inpatients simultaneously possessed information of a first major diagnosis for ALS and whole blood Pb concentrations, depending on the ubiquitous randomness of the medical data from hospital information system. Possible confounders were ruled out through choosing controlled inpatients. The controls were firstly selected from the same hospital, same department and same ward that treated ALS inpatients. Then, patients with other neurological diseases, like AD, PD, cerebral infarction, or dementia, were chosen as non-ALS group matched by age, gender, and admission time according to the radio of 1:9.

### Data preparation

The factors associated with ALS was analyzed and showed by Statistical Product Service Solutions (SPSS) 26.0, GraphPad Prism 8.0. Variables were presented as medians and interquartile (The first quartile was denoted as *Q1* and the third quartile was denoted as *Q3*) or frequencies and percentages, as appropriate. Blood index was analyzed independently using nonparametric pairwise Mann-Whitney tests. The correlations between metals/metalloids were calculated using Spearman method.

### Predictive analysis

**Fig. 1:**
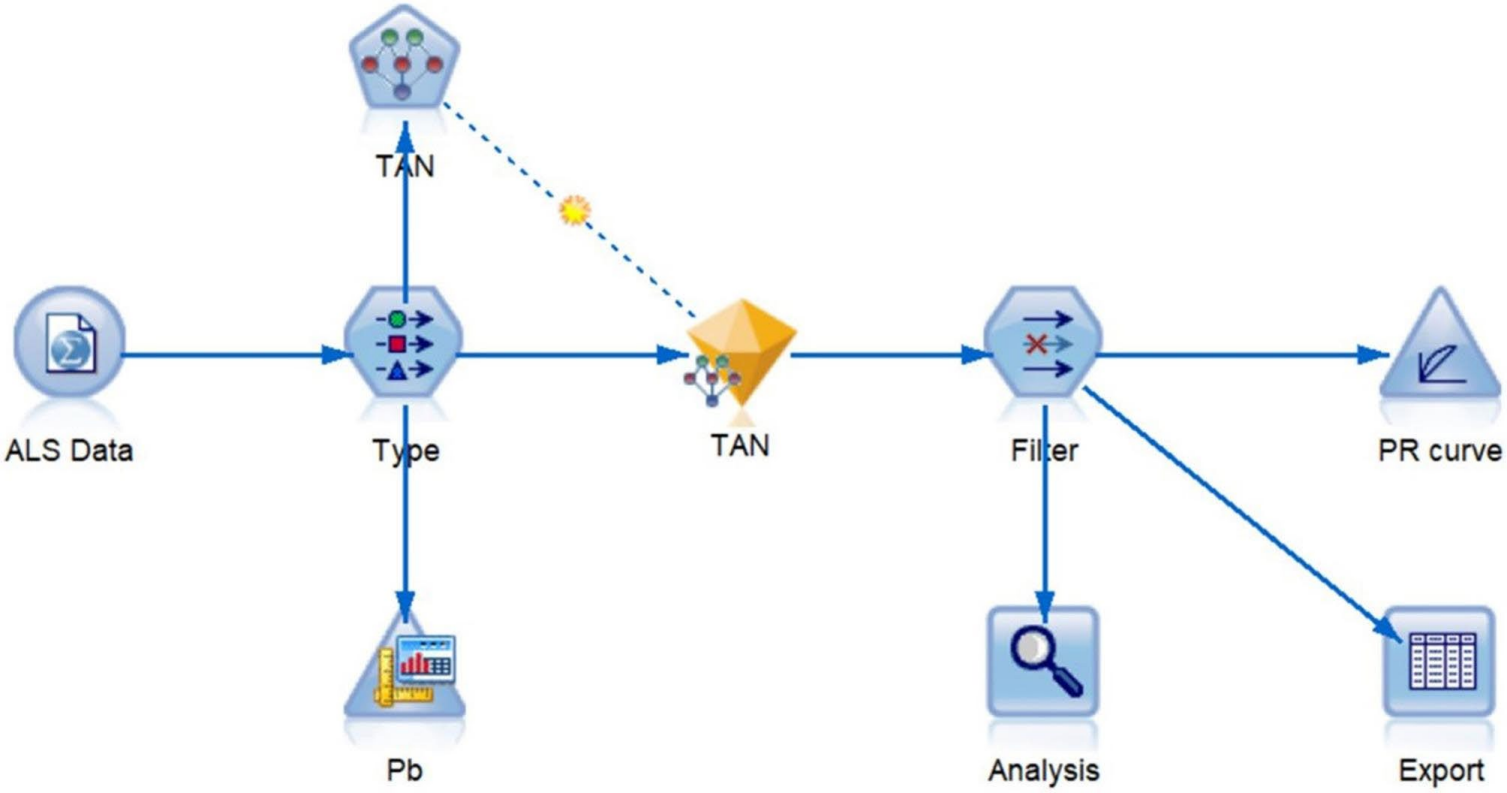
The detailed procedures of data analysis

The predictive model was established by SPSS Modeler 18.0. As a semi-naïve Bayes classifier, Tree Augmented Naïve Bayes (TAN), which allowed existences of correlations between predictive factors, was used to construct Bayesian networks. Precision ratio and recall ratio were used to draw a precision-recall (PR) curve. The specificity and sensitivity were comprehensive metrics that evaluated the performance of classification models ( [14]). A two-sided *p* value less than 0.05 was considered statistically significant. Figure 1 illustrates the detailed procedures of data analysis.

## Results

We identified 14 inpatients with a first diagnosis of ALS and 126 controlled inpatients matched by hospital, department, ward, age, gender, and admission time, hitting a total of 140 individuals as the study population.

### Demographic and clinical characteristics

The median age of the ALS inpatients was 59 years old (*Q1*: 48 years old, *Q3*: 64 years old). Male or female accounted for 57.14% or 42.86%, respectively. The demographic factors, especially the area of residence or occupational activity, did not show significant differences between ALS and controlled patients. Interestingly, in terms of site of onset, spinal-onset took the highest proportion, 87.40%, whereas bulbar-onset was 12.60%. Clinical characteristics of related lab tests results were analyzed. Total cholesterol and low-density lipoprotein (LDL) were significantly elevated in ALS cases compared to controls. In contrast, the control group had significantly higher levels of triglycerides and high-density lipoprotein (HDL) than ALS cases. In addition, uric acid, and creatine kinase were higher in the ALS group (Table 1).

**Table 1:** Baseline in patients’ characteristics

Characteristics	ALS cases(n = 14)	Controls(n = 126)	*p* value
Age(years)	59 (48–64)	57 (50–64)	0.84
Gender			
Male	8 (57.14%)	61 (48.41%)	0.78
Female	6 (42.86%)	66 (51.59%)	
Area of residence			
Urban	9(64.29%)	48(38.10%)	0.11
Rural	5(35.71%)	78(61.90%)	
Occupational activity			
Heavy	4(28.57%)	71(56.35%)	0.9
Light	10(71.43%)	55(43.65%)	
Site of onset			
Bulbar-onset ALS	1 (12.60%)		
Spinal-onset ALS	13 (87.40%)		
Lymphocyte	1.76 (1.24–2.28)	1.67 (1.41–2.04)	0.86
Neutrophils	3.27 (1.79–4.10)	3.04 (2.40–4.03)	0.74
Total cholesterol	4.42 (3.89–5.63)	1.20 (0.88–1.54)	< 0.01
Triglycerides	1.16 (0.93–2.32)	4.25 (3.61–4.84)	< 0.01
LDL	2.76 (2.19–2.96)	1.11 (0.95–1.37)	< 0.01
HDL	1.16 (0.99–1.32)	2.42 (2.03-3.00)	< 0.01
Serum albumin	40.50 (38.25–42.53)	39.15 (36.95–41.17)	0.27
Alanine aminotransferase	19.20 (13.25–29.35)	19.00 (13.55-24.00)	0.64
Aspartate transaminase	22.15 (17.20–31.00)	20.50 (16.95–22.45)	0.36
Creatinine	62.50 (51.73–75.25)	58.50 (50.00–67.00)	0.23
Uric acid	318.00 (244.25–361.00)	265.00 (221.25-308.75)	0.03
Creatine kinase	97.90 (50.88-195.33)	65.90 (27.65–87.03)	0.03
Glycosylated hemoglobin	5.80 (5.65–5.95)	5.70 (5.50–6.20)	0.55
Serum superoxide dismutase	147.45 (131.15-205.45)	166.05 (142.98–192.30)	0.63

Values are median (first quartile, third quartile) or counts (%)

### Comparisons of blood metal concentrations

**Fig. 2:**
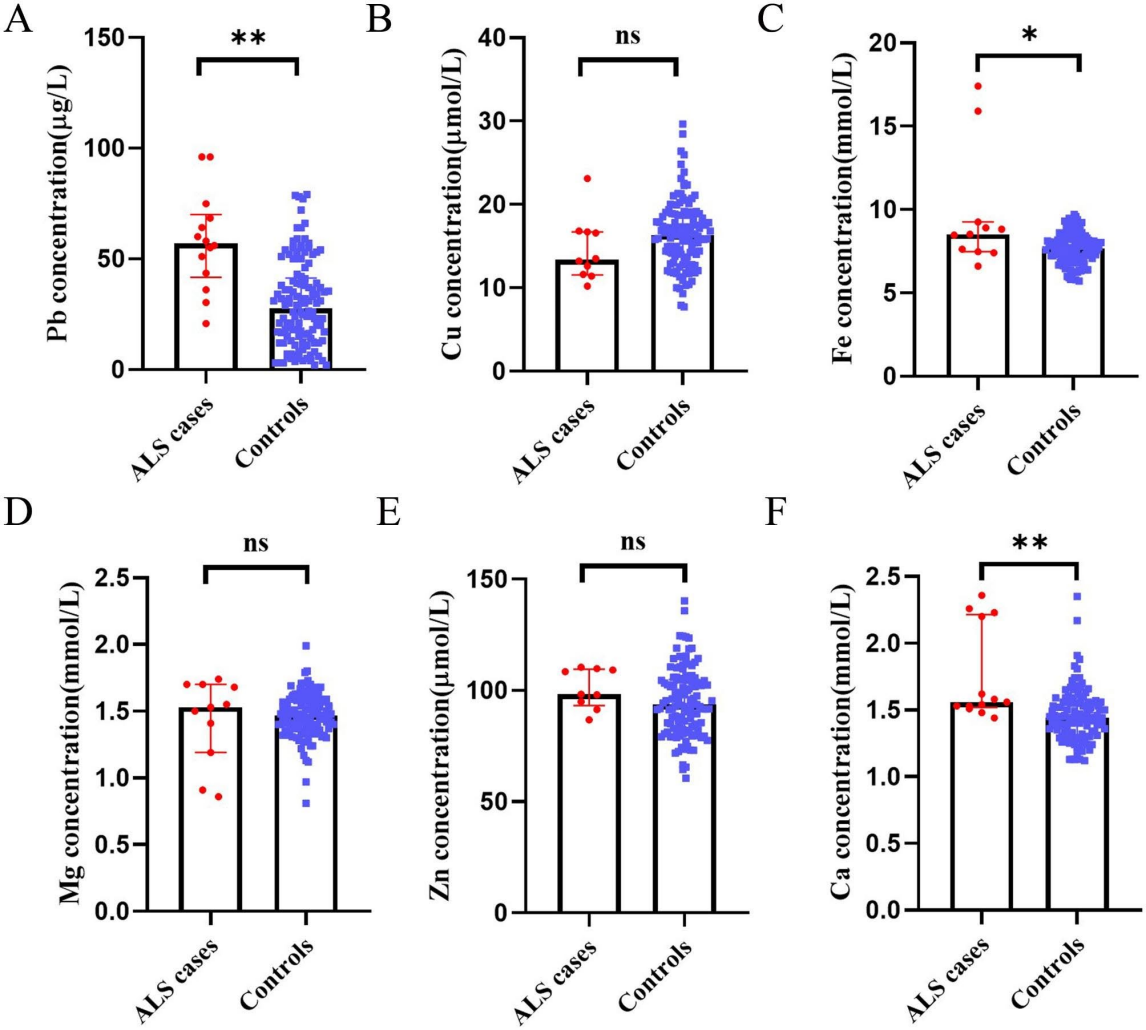
The comparisons of blood metal concentrations found in ALS cases and controls. **A**, **B**, **C**, **D**, **E**, **F** correspond to Pb, Cu, Fe, Mg, Zn, and Ca levels. Data presented indicate median and IQR. *: *p*-value < 0.05. **: *p*-value < 0.01. ns: not significant

**Fig. 3:**
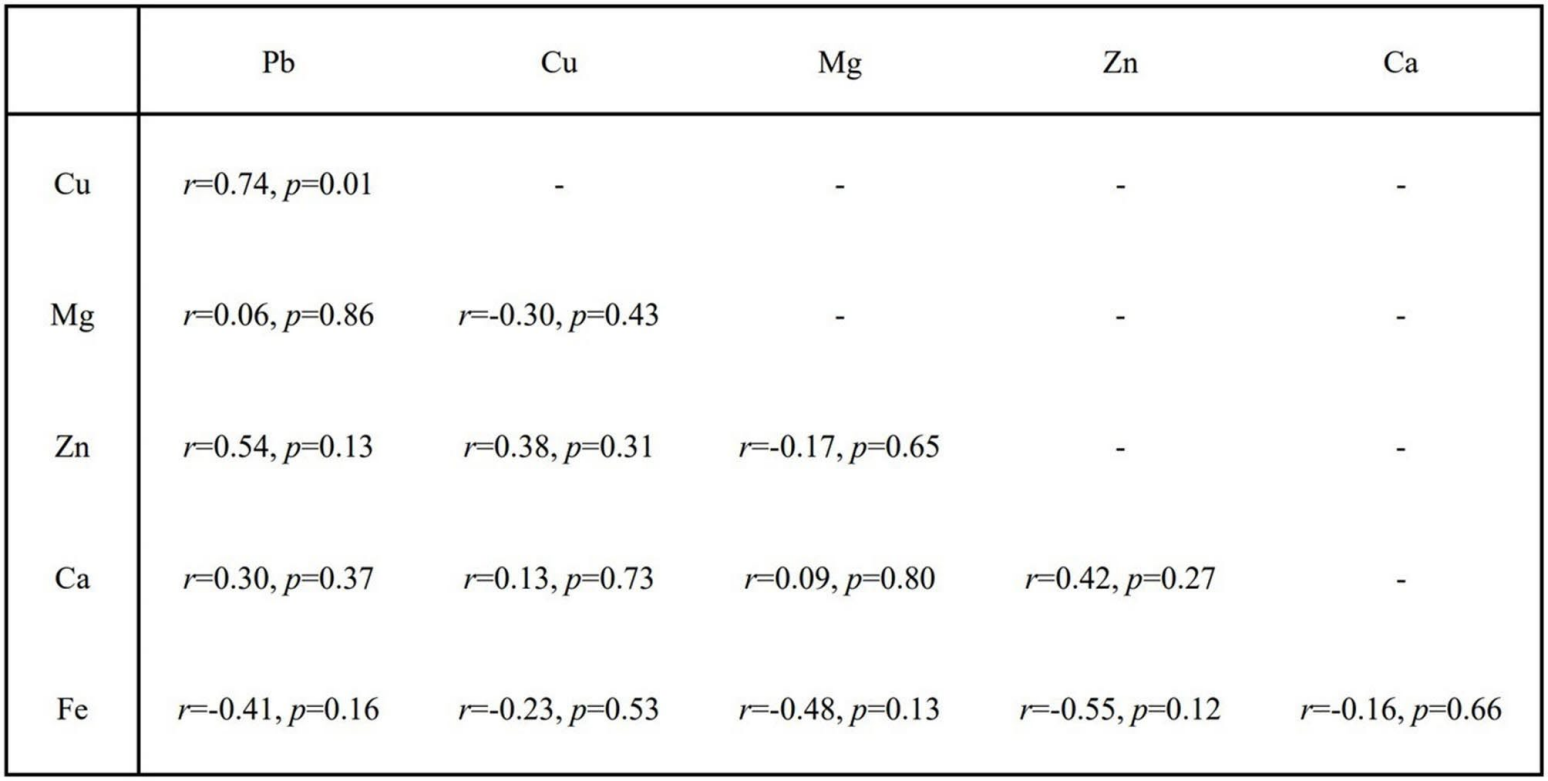
The pairwise correlation analyses for blood concentrations of Pb, Cu, Fe, Mg, Zn, and Ca

Median of blood Pb concentration of ALS was 57.00 μg/L (*Q1*:41.63 μg/L, *Q3*:70.01 μg/L), more than twice of the controlled group, 27.71 μg/L (*Q1*:13.75 μg/L, *Q3*:41.25 μg/L) (Fig. 2). Interestingly, we found blood Pb concentration of PD patients was 21.00 μg/L, nearly half of AD patients, 53.00 μg/L. The whole blood iron (Fe) or calcium (Ca) levels of ALS inpatients were 8.52 mmol/L (*Q1*:7.48 mmol/L, *Q3*:9.26 mmol/L), 1.56 mmol/L (*Q1*:1.52 mmol/L, *Q3*:2.22 mmol/L), slightly higher than non-ALS inpatients, 7.67 mmol/L (*Q1*:7.07 mmol/L, *Q3*:8.23 mmol/L), 1.44 mmol/L (*Q1*:1.31 mmol/L, *Q3*:1.55 mmol/L). However, the differences of copper (Cu), magnesium (Mg) and zinc (Zn) were not statistically significant (*p* > 0.05) (Fig. 2). Blood Pb concentrations were only positively associated with Cu (*r* = 0.74, *p* = 0.01), but not with Mg (*r* = 0.06, *p* = 0.86), Zn (*r* = 0.54, *p* = 0.13), Ca (*r* = 0.30, *p* = 0.37), and Fe (*r*=-0.41, *p* = 0.16) (Fig. 3).

### Model estimation

**Fig. 4:**
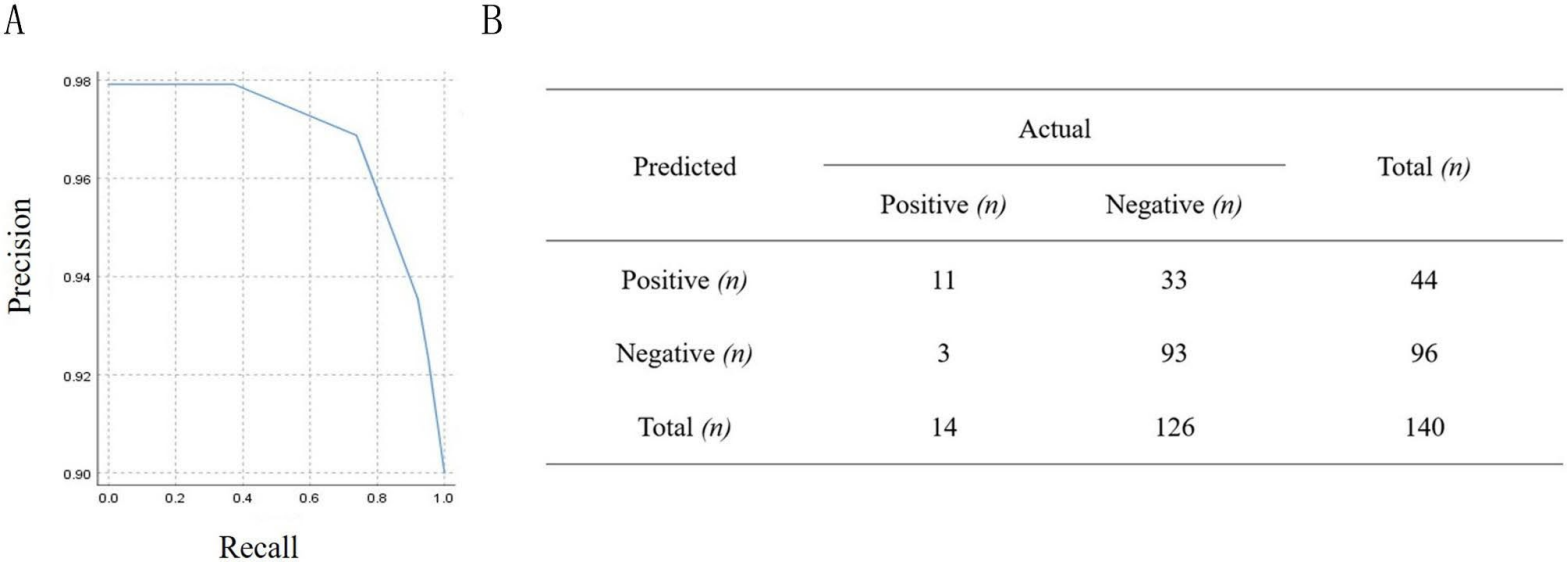
Prediction of ALS occurrence using blood Pb concentrations. **A**, **B** correspond to PR curves, diagnostic four-fold table

Here, a semi-naïve Bayes classifier, TAN, was used to construct the Bayesian networks. We focused on both statistically and biologically useful clinical variables including Pb, total cholesterol, triglycerides, LDL, HDL, uric acid, or creatine kinase. Among these factors, Pb, total cholesterol, triglycerides, LDL, HDL, and uric acid, showed the total coincidence rate in predicting ALS, reaching 90.00%, 92.14%, 92.86%, 80.00%, 92.14%, 90.00%, respectively. However, creatine kinase had low accuracy of only 19.29%. The specificity and sensitivity of Pb, total cholesterol, triglycerides, LDL, and HDL were both higher than 0.70, respectively. Interestingly, Pb combined with total cholesterol, as the co-input of Bayesian networks, showed a more comprehensive forecasting effect (Table 2). Here, we plotted a precision-recall chart as well as a confusion matrix to illustrate the performances of blood Pb levels in ALS prediction. The false negative rate was 21.42%, while the false positive rate was 26.19% (Fig. 4).

**Table 2:** Overall comparisons of TAN for different factors

Factors	Total coincidence rate (%)	Error rate (%)	Specificity	Sensitivity
Pb	90.00%	10.00%	0.79	0.74
Total cholesterol	92.14%	7.86%	1.00	0.90
Triglycerides	92.86%	7.14%	0.86	0.97
HDL	80.00%	20.00%	1.00	0.86
LDL	92.14%	7.86%	0.79	0.97
Uric acid	90.00%	10.00%	0.50	0.79
Creatine kinase	19.29%	80.71%	0.25	0.92
Pb and total cholesterol	92.86%	7.14%	1.00	0.89
Pb and triglycerides	94.29%	5.71%	1.00	0.50

## Discussion

ALS diagnosis is difficult in the early period because any upper or lower motor neuron signs may not be shown ( [7]). We have previously demonstrated that interleukin 2 (IL-2) and interleukin 6 (IL-6) may be used as an inflammation-related biomarkers for ALS severity ( [15]). In the present study, we investigated the predictive roles of blood Pb levels for ALS occurrence, and found that: (1) Pb concentrations in whole blood were significantly higher in ALS patients than in controls, almost twice as high; (2) Using blood Pb concentrations to calculate probability of ALS with Bayesian networks, TAN produced the total coincidence rate of 90.00%. The specificity, sensitivity for Pb was 0.79, or 0.74, respectively.

The history of research on the relationship between Pb exposure and ALS dated back to more than 100 years ago ( [16]). Case-control studies found higher Pb levels in ALS cases than controls in blood from a statistical view. However, statistically significant results were not observed for tissues like plasma/serum and cerebrospinal fluid (CSF) ( [5]). It has been proved that Pb tended to accumulate inside the erythrocytes rather than into the plasma component ( [17]). Simultaneously, Pb was captured by the choroid plexus or astrocytes, indicating its low concentrations in the CSF ( [18, 19]). This may explain the consistencies of whole blood Pb levels, compared with the plasma/serum or CSF.

Blood Pb levels were positively correlated with disease severity of ALS ( [20]). Consistent with previous findings, we observed that the concentrations of Pb in whole blood significantly increased by nearly twice in the ALS inpatients compared to the controls. Particularly, blood Pb concentrations were positively associated with Cu, but not other metals. This may derive from Pb-induced abnormal regulations of Cu transporters, like CTR1 and ATP7A ( [21]).

The mechanism underlying the interaction between Pb exposures and ALS is not completely understood. In zebrafish models, Pb exposure induced spinal cord motor neuron loss and ventral or dorsal motor neuron elongation changes ( [22]). The primary cultured mouse motor neurons were extremely sensitive to Pb exposure, and wild-type astrocytes in the co-cultured model failed to protect the damage, further suggesting that Pb has specific effects on the damage of motor neurons ( [23]). Our unpublished data provided abnormal aggregation of SOD-1 in motor neurons, damage of chaperone functions of GRP78 under Pb exposure.

Lack of data is a significant feature of medical data. Missing value replacements often resulted in offsets and errors. Bayesian networks were built upon a strong foundation in causality and probability theory, regardless of the missing values ( [24]). Compared with other machine learning methods, like artificial neural network, logistic regression, support vector machines, k-nearest neighbor algorithm, Bayesian networks produced best results in predicting the ALS with blood indexers ( [11]). Using this model, we took blood Pb concentration as a preferable biomarker of ALS, because of its properties of exogenous substances. To excluded Pb’s predictive roles of other neurodegenerative diseases, like PD, AD, we brought total cholesterol or triglycerides into this model to enhance the specificity. Previous studies have demonstrated the benefits of lipid-rich diet in slowing disease progression in ALS patients ( [25]). Patients with elevated triglycerides levels and LDL/HDL ratio extended survival by almost one year ( [26, 27]). Total cholesterol was positively associated with the risk of ALS ( [28]). Some groups reported that TDP-43, pathological hallmark and one of the causal genes for ALS, regulated cholesterol metabolism via sterol regulatory element-binding protein 2 (SREBP2) ( [29, 30]). These results may provide a smart and simple strategy with great clinical prospects for identifying patients at high risk of ALS at an early period.

The study has some limitations. First, there may exist selection bias of ALS cases who received blood Pb test because these patients tended to report their history of Pb exposure. Second, we did not compare the Pb levels between the whole blood samples and plasma/serum or CSF. Third, factors of mental or physical check and gene analysis associated with ALS were not considered in this study. Fourth, prospective studies with large samples should be designed to validate its accuracy of this model.

In conclusion, based on the medical record from hospital information system, blood Pb levels of ALS was more than twice as high as the controls. Using blood Pb concentrations to calculate probability of ALS, Bayesian networks produced ideal results with high accuracy. The study highlighted the predictive roles of blood Pb concentrations on ALS occurrence and developed a forecast model to help identify ALS patients with high risk.

## Data Availability

The data that support the findings of this study are available from PLAGH, but restrictions apply to the availability of these data, which were used under license for the current study, and so are not publicly available. Data are however available from the authors upon reasonable request and with permission of PLAGH.

## Abbreviations

AD: Alzheimer’s disease
ALS: Amyotrophic lateral sclerosis
C9ORF72: Chromosome 9 Open Reading Frame 72
CSF: Cerebrospinal fluid
fALS: Familial ALS
GRP78: Glucose-regulated protein of 78 kDa
HDL: High-density lipoprotein
IL-2: Interleukin 2
IL-6: Interleukin 6
LDL: Low-density lipoprotein
Pb: Lead
PD: Parkinson’s disease
PR: Precision-recall
sALS: Sporadic ALS
SOD1: Superoxide Dismutase 1
SPSS: Statistical Product Service Solutions
SREBP2: Sterol regulatory element-binding protein 2
TAN: Tree Augmented Naïve Bayes
TDP-43: TAR DNA-binding Protein of 43 kDa
